# The Institutional Worker

**Published:** 1913-03-29

**Authors:** 


THE
### INSTITUTIONAL WORKER
**Being a Special Supplement to "The Hospital"**

### A Laundry Bill.

THE estimated cost per head per week in this institution, which is a consumptive sanatorium, is as follows: Soap, yellow (3 lb.), 9½d.; soap, carbolic (1 lb.), 3½d.; soda (18 lb.), 6d.; blue, 1d.; starch, best (¾ lb.), 4d.; borax, 1d.; bleaching soda, 1d.—total material, 2s. 2d.; wages per week, two maids, 13s. 3d.; coal and coke, airing, 5s. 6d.; total £1 0s. 11d. The cost per head is 8d. There are twenty-four patients and eight staff. The laundry is done on the premises by steam machinery.—IOTA.

[We consider the results you are able to show are highly creditable, and the cost extremely low.—ED.]

### The Control of Breakages.

THE difficulty you experience in regard to the common "accident" is one often felt, and arises out of the ignorance and heedlessness of the inexperienced workers you are compelled to trust. Where many people are handling the same things in rapid succession, there must necessarily be a larger amount of loss by breakage; but it should be possible to fix a given amount that will represent wear and tear, and then to aim at keeping within that limit. An organised system of checking is a necessity, and there is no part of institutional routine that will produce better results or be more productive of true economy than this. By making each person responsible for some part of the property, and then regularly checking it, even the most junior members may do service, and as the art of training is to cultivate a sense of responsibility a great deal has thereby been achieved. The heads of different departments should have definite days for inspecting various articles in use—table crockery, ward utensils, etc. Those who use them should be furnished with written lists, and made responsible for keeping the number correct. In this way the newcomer can quickly be trained to recognise her share in the responsibility. The youngest maid will take a pride in having her numbers correct, and articles carefully arranged, if she can be quite certain that on the expected morning the one in charge will be ready to count them.—C. E. A.

### Checking the Losses.

ONE day in the week or month, as convenient, should be fixed for making good all losses, and rigidly adhered to. The broken or worn article should be sent for inspection (and the pieces retained), and no loss made good until it has been produced. A trained observer will thereby be quickly capable of judging whether there has been fair usage. A hair brush showing burnt bristles, a linen article minus any trace of mending, rubber goods with sides adherent to each other, or an enamel article chipped and cracked in all directions will tell their own tale. There should be some rule for bringing home carelessness, such as a fine for part payment; but, whatever plan be made, it should not be too drastic. Much loss, it is true, arises from heedlessness and inexperience, but rarely from deliberate carelessness. The younger members of a staff can be greatly helped by being taught the cost of many of the articles they use.—C. E. A.

### OUR BUREAU OF INFORMATION.

### Rules for Correspondents.

1. Every letter, on whatever subject, must be accompanied by the coupon to be cut from the back cover (inside page) of THE HOSPITAL, current issue, and must contain the name and address of the correspondent with pseudonym for publication if desired.
2. Replies by post cannot be given, save under exceptional circumstances at the Editor's discretion.
3. Letters from Approved Homes in reply to special needs published in the Bureau should state terms and full particulars, and be sent prepaid under cover to the Editor of the Bureau with name written across coupon for identification.
4. Proprietors of Homes which have not yet been entered on the List of Approved Homes, but have spare accommodation likely to suit special needs, are invited to write for an application form for registration. The fee for registration, which includes two announcements of the Home in the Bureau and other privileges, is 10s.
5. All communications to be addressed to the Editor of THE HOSPITAL, 28 Southampton Street, Strand, London, W.C., and marked "Bureau of Information."

### Approved Homes.
#### Important Notice.

IN consequence of the large amount of correspondence involved in registering Homes, forwarding letters, and communicating with persons in need of accommodation, the Editor is compelled to raise the fees which have hitherto prevailed in this department. After April 1 the registration fee for admission to the Approved List of Homes will be Ten Shillings, to cover also two announcements in the Bureau, and to include the use of the Bureau for forwarding letters for one year from the date of the second announcement of the Home. After the first year the principals of Approved Homes may continue to have their letters forwarded at a charge of Five Shillings a year. All who have received papers, but have not yet sent them in, should forward them not later than Monday, March 31, in order to be admitted to the List at the old rate.

The special needs of any who are sick or in difficulties can be made known in these columns free of charge.

#### Home Wanted.

THE patient is an elderly lady requiring care. She has been under asylum treatment for several years, her ailment being psychical epilepsy, which recurs periodically, leaving often calm intervals lasting some weeks. She is never violent, and would not be "certificated." Only very moderate terms could be paid. Replies to A. W. M. (157A).

#### Partly Paralysed Maid.

THE patient has had a stroke while on duty at a boy's school, and remains partly paralysed on one side, with speech badly affected. We strongly recommend you, when she leaves the Cottage Hospital, to place her where she could have the advantage of further treatment, with massage and electricity. Apply to the Secretary, West End Hospital, 73 Welbeck Street, London, W. If a small sum can be contributed weekly it may facilitate her admission, but, as she has no friends or relative able to help, any little

### Training at Thirty-five.

THERE are a good many provincial institutions in which the age limit extends to 35. Among these are the Bury General Infirmary; the Chichester General Infirmary; the Coventry and Warwickshire Hospital; the Victoria Hospital, Folkestone; the Hertford County Hospital; the Kidderminster Infirmary; the Royal Hospital, Richmond; the Mill Road Infirmary, Liverpool; and the Parish of Portsmouth Infirmary, Milton. As you hold the C.M.B. certificate, are strong and active, and have good nursing experience, perhaps one of our readers may offer you a chance of getting the period of regular training for three years which you desire. Get your testimonials and certificates typed and enclose copies of recent recommendations from two persons in good position. Letters forwarded to M.F.P.

### Immediate Admission to Sanatorium.

AMONG the least expensive establishments are the Kelling Sanatorium, Holt, Norfolk, 30s. a week; the Maitland Sanatorium, Peppard Common, Oxon, 30s.; the Coppins Green Sanatorium, Clacton-on-Sea, for clerks and artisans. No doubt you know of the Ransom Sanatorium, Sherwood Forest, for residents in your county. We fear there is likely to be a considerable delay in many similar cases to your own in getting the sanatorium benefit of the

Insurance Act into smooth working order.—SANT.

### Nursing in Tasmania.

YOU must fully understand that this is an agricultural country, with no large proportion of people able to pay at a high rate for the ministrations of a nurse in sickness. The population of Hobart, the largest town, is under 40,000, and on arrival, unless you are prepared to take up some domestic post, you may find yourself without employment for a considerable time. Unless you are going out to friends with a view to emigration, we should certainly counsel you against the voyage.—ESPERANZA.

### Waste of Gas.

THE first step towards checking waste is to read the meter regularly every day, and note the amount of gas used during the twenty-four hours. If you have not yet accustomed yourself to do this, ask the gas company to send you an official to explain the system. You may easily ascertain whether there is a leakage by turning off every tap and then noting whether the hand on the smallest dial remains perfectly stationary. Reduce the pressure of gas by turning it half off during the day-time, so that no unnecessary quantity is used at rings or burners. Take note whether the cook allows the gas to flare and roar. Again, take particular heed that the little holes close to the taps on the stove are perfectly clean. Heat is obtained by the mixture of gas which you pay for and air for which you pay nothing. If the holes are choked up you obtain less heat, and you use more gas with too little air. Few domestics will trouble about these details unless the reasoning is explained to them. Old lighting burners on brackets or bad burners are responsible for much waste and a poor light. It saves expense to have them periodically overhauled and renewed.—ASSISTANT.

### Browning for Gravies.

THE difficulty you mention about the gravy is chronic in institutions. It is as you say very expensive to buy browning, and it takes a long time to get the right colouring effect by frying meat and flour. You should make caramel browning, or browned flour, and get it in stock. For the caramel, put 1 lb. of Demerara sugar into an old untinned iron saucepan. Melt it over the fire until it becomes a very dark brown, almost black, stirring it now and then. Draw the pan to the side and pour in half a pint of water. It will make a great noise and the sugar re-harden, but continue to boil and stir it until the syrup is rather like thin treacle. When done let it cool down, pour into a dry bottle, and use as required. To brown flour spread 1 lb. on a large flat tin. Put it in a moderately hot oven until it is a bright brown colour. Pass it through a wire sieve, put it when cold into air-tight tins, and use for thickening instead of white flour.—E. H. D.

subscription will be needed many times over before she is well. Being a hopeful case, she might obtain admission to any of the hospitals for diseases of the nerves.—E. M. L.

### Help in Maternity Training.

THE London County Council offers scholarships periodically for midwifery training, and so do many of the County Associations. Study the advertisements. Write to Miss Pritchard, Lady Superintendent, District Nurses' Home, Howards Road, Plaistow. The terms are very moderate, and the training admirable.—MRS. K. W.

### To Obtain Sanatorium Post.

THERE is much competition for obtaining the secretaryship of the newly formed sanatoriums under the Insurance Act, and there is no short cut to success. As the person to whom you allude has had experience in this particular line, and possesses good testimonials, there is everything in his favour. The appointments are advertised in the medical and local papers from time to time, and application must be made as directed. But great caution is indicated in relinquishing a certainty for the chances of open competition. —M. W. B.

### Training at Twenty-one.

YOU are too young to begin regular training. Write to the Matron, Babies' Castle, Hawkhurst, Kent, and ask for particulars of their course. Also get a copy of "How to Become a Nurse," price 2s. 6d. (The Scientific Press). It will give you full information about outfit and the best way of getting trained. We should advise your filling up another year or two in some small institution, and then joining the Poor-Law service by entering as probationer at a Poor-Law infirmary.—E. S., Hove.

### Nursing of General Paralysis.

THE patient should be removed from home, and the daughters who are at present on the verge of breaking down should be relieved of all strain without delay. The cerebral excitement feared as a consequence of removal is probably greatly exaggerated by the relatives. Since the doctor makes no objection, a Home should be selected in the vicinity, and the change of quarters could be effected while the patient is under a sleeping draught. The well-being of your daughters ought, in our judgment, to be placed first, especially as the sufferer is not in a condition to experience more than a passing inconvenience from the change.—WORRIED FATHER.

### Welfare Workers.

As a trained nurse you would stand a very good chance of obtaining work in this direction, but you must show that you add to your other qualifications some knowledge of social conditions among the poor. The posts are usually under firms of employers who have a large staff of female workers, and the work embraces not merely matters affecting the well-being of the staff, but also the aid of their relatives by advice, and sometimes by practical benevolence. As the work is in its infancy, every welfare worker is to some extent a pioneer and has to find out in what direction her activities may best bear fruit. In large establishments high salaries are occasionally given to the heads of these departments, but as a rule from £50 to £100 would be the limit. Ascertain what factories or business firms in your neighbourhood employ workers of this description, and offer your services, should vacancies occur, with a copy of typed testimonials.—NURSE C.

### Rich Suet Pudding.

IF your patients are beginning to revolt against so much bacon and oil, try the following recipe for tempting them to consume fat: Put 6 oz. of flour into a basin with half a teaspoonful of baking-powder, the same of salt, and 2 oz. of white crumbs. Add 5 oz. of finely chopped beef suet. Mix all to a soft, rather sticky dough with sweet or sour milk. Put this mixture into a well-greased basin, twist a bit of greased paper over the top, and steam it for three hours. Serve with butter and Demerara sugar. —SANATORIUM.

### Fish Macaroni.

THIS is an excellent Friday dish, and may relieve the monotony of which you complain. Take sufficient cod for your party and partly boil it, removing skin and bones. Break up the macaroni in pieces and boil it till tender. Mix well together with four ounces of grated cheese and season with pepper and salt. It should then be put into a fireproof dish and covered with grated cheese and breadcrumbs, with little dabs of butter. Bake in a moderate oven till brown.—SISTER F.

### The Editor's Letter-Box.

### Communications have been received and attended to from:—

Mr. A. W. M., Putney; Mr. W. M., London; Sister E. P., Clacton-on-Sea; Nurses McM. and McG., Strathaven; Miss J. H.; and others.

NURSE E. C.—Very glad to know that you are able to receive the patient we sent to you who wants accommodation in your neighbourhood.

ELIZABETH. — Many thanks. We are always glad to see your handwriting.

MRS. PIKE.—We are unable to place patients in communication with any but Homes on our Approved List.

J. B.—Glad to have your change of address and to know that the help you receive through the Bureau enables you to choose a better room.

### Editor's Notices.

**Contributions:**

Contributions should be written, or preferably typed, on one side of the paper only, and all articles sent in are accepted upon the distinct understanding that they are forwarded to THE HOSPITAL only.

The Editor cannot undertake to return MSS. not used, but when a stamped directed envelope is enclosed the MSS. may be returned if a special request is made.

Accepted articles and paragraphs of news will be paid for after publication at the scale rate.

**Address:**

To prevent delay all contributions and letters on editorial business must be addressed exclusively to the Editor, "The Hospital," 29 Southampton Street, Strand, London, W.C. It is important that this regulation shall be strictly observed.

**Correspondence:**

Correspondence on all subjects is invited. The name and address of correspondents must be given as a guarantee of good faith, but not necessarily for publication.

**Special Articles:**

Special articles are invited, and questions, inquiries, and paragraphs upon all matters relating to the work, administration, and management of general, special, mental, fever, cottage and convalescent hospitals, sanatoria, homes, institutions, societies and organisations for the treatment or care of the sick, injured and dependants of all classes. Every matter affecting the interests and welfare of the staff of all grades working in these institutions will receive special consideration.

**Administrative Medicine, Research and Items of News:**

Special terms will be paid for approved articles on subjects in this wide field by experts who have made some section of it their special study and interest. We wish to give prominence to every movement tending to advance accuracy of diagnosis, efficiency in treatment, and the development of modern methods to eradicate disease and promote the welfare of the suffering.

**A Bureau of Information:**

We invite inquiries and applications for information and help in providing for that numerous class of sufferers who require special care or house-room which they cannot obtain in their own homes or secure for themselves. We cannot, however, prescribe, or recommend practitioners.

**Books for Review:**

Publishers are particularly requested to send advance proofs of any new books of importance whenever possible, as the Editor has made arrangements to publish immediate reviews on a new plan.

**Photographs, Plans, Blocks, and Illustrations:**

It is requested that wherever possible MSS. may be accompanied by illustrations in any of the above forms. The name of the sender and of the article to which it belongs should be written on the back of each photograph, drawing, or block for purposes of identification.

**Local Papers:**

Newspapers containing reports or news paragraphs should be marked and addressed to the Sub-Editor.

**The Coupon System:**

A coupon will be found at the bottom of the third inside page of the cover each week. This coupon must be attached to every question or inquiry to which an answer is desired in THE HOSPITAL.

### Next Week's Special Number.

THE entire edition of the last Special Number of THE HOSPITAL was sold out by the Friday evening, and in consequence of the valuable information it contained it was quite out of print within a few days of publication. This is due undoubtedly to the high appreciation in which THE HOSPITAL is regarded by Executive Officials and Institutional Workers of all grades throughout the country. No Journal deals so adequately and comprehensively with Institutional affairs, and consequently each issue is eagerly awaited by all keen and progressive Officers in the Charitable, Poor-Law, and Municipal Services. Those desirous of receiving THE HOSPITAL regularly should remember that the Journal is on Sale on Thursday in each week, and that it is essential to place their order permanently with their Newsagent and Bookseller, or send a subscription direct to the Publishing Offices at the undermentioned rates.

**Subscriptions** may begin at any time, and are payable in advance. Cheques and Post-Office Orders should be crossed London County and Westminster Bank, Covent Garden Branch, and made payable to the Manager of THE HOSPITAL.

RATES OF SUBSCRIPTION (payable in advance).

| | | |
|---|---|---|
| **UNITED KINGDOM.** | | |
| Three Months (including Postage) | ... | 2s. 0d. |
| Six Months | do. | 4s. 0d. |
| Twelve Months | do. | 6s. 6d. |
| **FOREIGN AND COLONIAL.** | | |
| Three Months (including Postage) | ... | 2s. 6d. |
| Six Months | do. | 5s. 0d. |
| Twelve Months | do. | 8s. 8d. |

*SUBSCRIPTION FORM.*

*Please send me* THE HOSPITAL *for..............months, commencing with your issue of.........................., for which please find enclosed..........................*

*Name*..........................................

*Address* ..........................................

..........................................

*Date*..................................

*To* .................................... *Newsagent.*

Or to

*The Manager,*

THE HOSPITAL,

The Hospital Building,

28/29 Southampton Street,

Strand, London, W.C.

### Manager's Notices.

**Letters relating to the publication, sale, and advertising departments of "The Hospital" must be addressed to The Manager, "The Hospital," The Hospital Building, 28 and 29 Southampton Street, London, W.C.**

**Advertisements:**

To ensure insertion, all advertisements for THE HOSPITAL must reach the Manager not later than Wednesday morning in each week. For scale of charges see pages v and 4.

**Remittances:**

**It is especially requested that remittances be made by Cheque or Postal Order, and in halfpenny stamps only when the amount is under sixpence.**

### Books on Hospitals and Institutions.

By SIR HENRY BURDETT, K.C.B., K.C.V.O.

### HOSPITALS & ASYLUMS OF THE WORLD.

In Four Volumes, with Portfolio of Plans. Complete, £12 12s.

Their Origin, History, Construction, Administration, Management and Legislation; with Plans of the chief Medical Institutions, accurately drawn to a uniform scale.

Vols. I. and II. (only).—Asylums and Asylum Construction, £6 15s.
Vols. III. and IV.—Hospitals and Hospital Construction—with Portfolio of Plans, £9.
The Portfolio of Plans (20in. by 14in.), separately, price £4 14s. 6d. Carriage extra.

IMPORTANT.—AS ONLY A FEW COMPLETE COPIES OF THE WORK REMAIN UNSOLD, Secretaries and Institution Librarians are recommended to take immediate steps to procure this Encyclopædia Britannica of Hospital and Institutional affairs.

### BURDETT'S HOSPITALS & CHARITIES.

The Year Book of Philanthropy, and Hospital Annual.

"The ideal of what a work of reference ought to be."—*The Times.*

Published Annually, over 1000 pages. 10s. 6d. net; 10s. 11d. post free.

This Annual is now in its Twenty-fourth Year, and the complete series orm a Library of Hospital Facts and Figures absolutely unobtainable from any other source. Everyone associated with or interested in our Hospitals and Charities should not only obtain the new edition, but all previous issues, since they are a valuable history of the Hospital movement throughout the English-speaking world.

### COTTAGE HOSPITALS, GENERAL, FEVER AND CONVALESCENT.

Their Progress, Management and Work in Great Britain, Ireland and the United States.

Third Edition, profusely illustrated, with nearly 50 Plans, Diagrams, etc.
Cloth gilt. 10s. 6d. net.

A New and Abridged Edition of this book is now in the press and will be published shortly.

### THE UNIFORM SYSTEM OF ACCOUNTS.

For Hospitals and all Classes of Public Institutions.

With Special Forms of Accounts and Specimen Rulings of a complete set of books.
New Edition. In the Press.

### ACCOUNT BOOKS FOR INSTITUTIONS.

Designed in accordance with the Uniform System.

(Full description of Books and Prices on application.)

### MEDICAL ATTENDANCE OF LONDONERS.

An Economical System of Medical Relief freed from existing abuses and adequate to protect all classes.

In paper cover. 1s. post free.

### HOW TO BECOME A NURSE.

The Nursing Profession. How and Where to Train.

A Complete Guide to Training for the calling of a Nurse, with particulars of Nurse Training Schools in the United Kingdom and Abroad.
Price 2s. net. By Post, 2s. 4d.

"The advice to women desirous of becoming Nurses is simple, sound and discreet."—*Lancet.*

Of all Booksellers, or of

**The Scientific Press, Limited,** 28/29 SOUTHAMPTON STREET, STRAND. LONDON, W.C.

